# Animal navigation without mental representation

**DOI:** 10.1007/s11097-023-09940-z

**Published:** 2023-10-07

**Authors:** Bas van Woerkum

**Affiliations:** Centre for Cognition, Culture and Language, Faculty of Philosophy, Theology and Religious Studies, Radboud University, Nijmegen, The Netherlands

**Keywords:** Animal navigation, Wayfinding, Cognitive map, Ecological psychology, Vista, Affordances

## Abstract

Do animals require rich internal representations, such as cognitive maps, to navigate complex environments? Some researchers believe so, as they argue that sensory information is “too poor” to account for animals’ wayfinding abilities. However, this assumption is debatable, as James J. Gibson showed. Gibson proposed that wayfinding involves detecting information about environmental structure over time and used the concepts of “vistas” and “transitions” to explain terrestrial navigation. While these concepts may not apply universally to animal navigation, they highlight the importance of exploiting stable environmental structures for wayfinding. By searching for species-relative environmental structures, we may gain insight into the navigational abilities of different nonhuman animals, while recognizing the unique evolutionary histories and ecological contexts that have shaped these abilities.

## Introduction

**Fig. 1:**
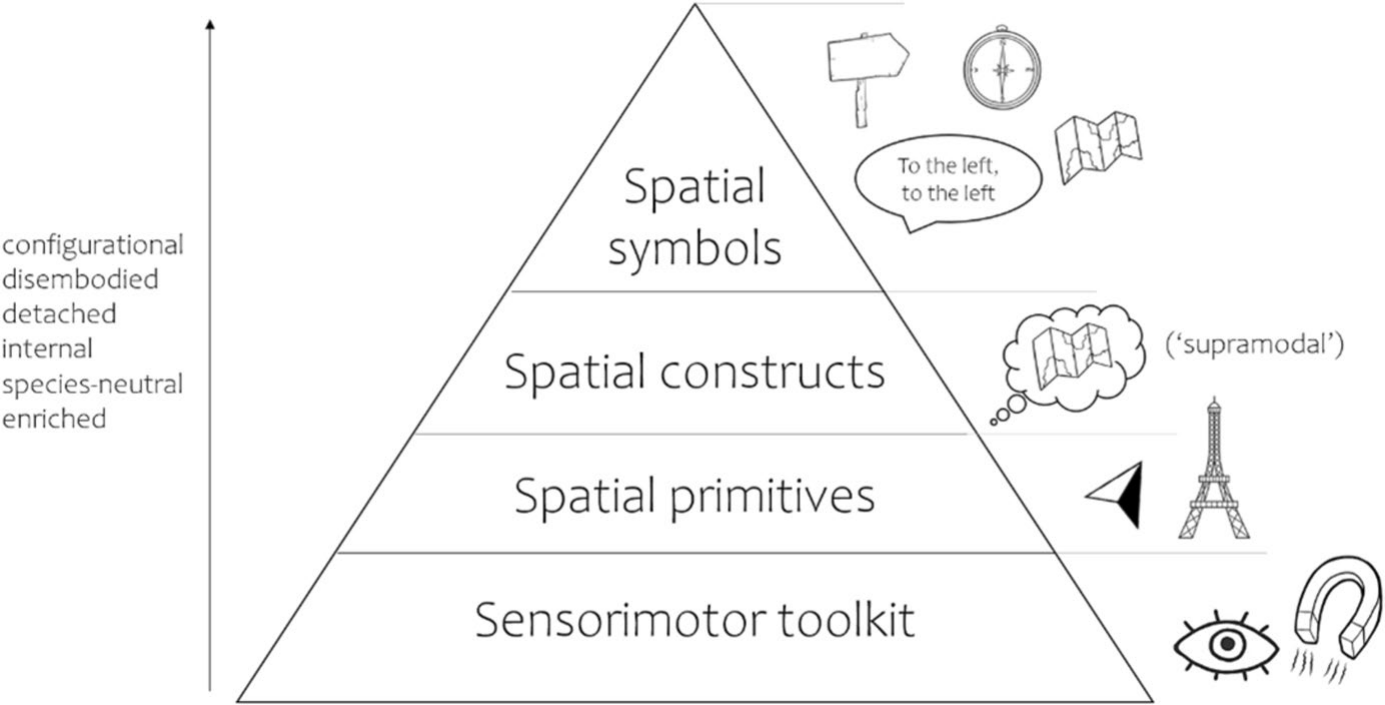
The Navigation Toolkit (Wiener et al., 2011). The sensorimotor toolkit contains basic skills of sensing and acting, like visual perception and magnetoreception. The spatial primitives include tools like compass heading and landmarks. The spatial construct level lists, most prominently, cognitive maps. Spatial symbols are defined as uniquely human. This level includes wayfinding signage, actual maps, verbal directions and compasses. As perceived complexity increases, the tools that animals employ seem to become more and more detached from the specific action-perception systems of that animal

When an albatross lands on a nearby island, she exhibits a simple form of navigation known as beaconing. However, albatrosses also voyage great distances and return to their home island, demonstrating a more complex navigational ability. What constitutes the difference between these two behaviors and what factors contribute to the perceived complexity of the latter feat? A renowned group of animal cognition researchers have proposed an answer to this question: the perceived difference is due to *representational* complexity (Wiener et al., 2011). To compare the navigational abilities of various species, Wiener et al. (2011) present a “navigational toolkit”, which has four levels, ranging from low to high: the sensorimotor toolbox, spatial primitives, spatial constructs, and spatial symbols (unique to humans) (see Fig. 1). Each higher level builds on tools of the lower level, and higher-level tools encode the world in increasingly *configurational* ways (Heft, 2013).

The term “configurational” captures a core tenet of the representationalist perspective, as it views cognition as a process of isolating and internally representing aspects of the environment, and then linking and combining these to construct more complex representations.^1^ Researchers who think of animals as configurational knowers believe that animals—or their brains—run internal manipulations over representations of the environment. Their brains may calculate distance based on visible landmarks, for instance. As configurational knowers, animals are “detached from the landscape and disembodied rather than being immersed in it” (Heft, 2013, p. 268). Moreover, when animals voyage beyond what they can currently see, hear, smell, or feel, representationalists seem to think of them as even more configurational, disembodied knowers.

Gibson and Gibson (1955, see also Gibson & Pick, 2000) used the term “enrichment” to describe theories, such as Wiener et al.’s (2011) toolkit, that advocate increasing abstraction from the environment. Enrichment thinkers assume that the information provided by perceptual stimuli to the perceptual systems is too poor to explain complex feats of navigation or wayfinding.^2^ Because the information is too poor, it needs to be internally enriched—supplemented by memories, expectations, concepts, and other representations—to make navigational feats higher on the ladder possible. Gibson (1979/2015) disagreed that the information is too poor, and introduced alternative concepts for explaining navigation: most notably, “vistas” and “transitions”. He employed these concepts to show that, at least for earth-dwelling humans, perceived navigational complexity doesn’t necessarily entail enrichment and abstraction from the environment (see also Gibson & Pick, 2000, p.10).

Inspired by Gibson’s concepts, I will argue that we can better understand how nonhuman animals’ navigate by examining how they learn to perceive the environment’s nested structure over time. This alternative view ties navigational ability to the perceptual systems and environments of animals, even in cases of perceived complexity. As such, it fits better within an evolutionary perspective, or more specifically, the view that brains, and the cognition they support, evolved in tandem with a specific body to enable the control of action in dynamic ecological niches, rather than to harbour specific cognitive mechanisms or tools to solve the “problems” of navigation (see Barrett et al., 2022; Barrett et al., 2007; see also Keijzer, 2015).

I will first discuss, in more detail, the enrichment view: the view that animals utilize landmarks and relations between landmarks as a form of configurational knowledge (Sect. 2). After offering some reasons to consider an alternative (Sect. 3), the remainder of the paper will explore the view that animals learn to perceive their environment’s nested structure over time. I start by introducing Gibson’s concepts for terrestrial wayfinding (Sect. 4). Then, I will discuss navigation over the oceans, to show that more dynamic features, such as winds and waves, provide stability and structure to a trained perceiver, and extract some general features of wayfinding, i.e. not limited to terrestrial, vision-based navigation (Sect. 5). After that, I discuss how albatrosses find their way around by relying on the oceanic scentscape (Sect. 6.) and how they are able to reach their destination without representing these destinations (Sect. 7). I end with some general implications for research.

## Navigation and enrichment

Animals, humans among them, employ various skills to navigate their environments. This includes beaconing, route following and path integration (Rescorla, 2018; Wiener et al., 2011). An animal that approaches a feature—say a tree—within the field of view by fixating on it, is beaconing. An organism that retraces a series of remembered turns is route-following—for instance, turning left when you meet the big oak tree and continuing straight ahead until you encounter the face-shaped rock. There’s no need to know *that* you’re on a particular route. Human beings can navigate familiar terrain while thinking about what to make for dinner. Path integration requires an organism to keeps track of a starting position by monitoring distance and direction travelled. The organism could rely on a step-counting mechanism (e.g. desert ants, see Wittlinger et al., 2007), optic flow patterns, or something else. In the navigational toolkit, level-two elements, such as landmarks, compass heading and speed and acceleration monitors, enable these skills (Wiener et al., 2011).

Higher on in the toolkit we find cognitive maps. Rescorla (2018) calls the cognitive map the “most controversial one” of navigational strategies in the field of animal navigation (p.34) and obscurity about the representational nature of cognitive maps surely contributes to the controversy.^3^ The term “cognitive map” was originally introduced by Tolman (1948) and is generally described as a mental representation that encodes information about landmarks and geometric relations among landmarks, as well as one’s own position, in an allocentric way. Rescorla (2018) distinguishes maps in the loose sense from maps in the strict sense (see also Rescorla, 2009). Loosely understood, a cognitive map is a “mental representation that represents geometric aspects of the environment” in a metric (distances, angles), topological (connectedness and adjacency) or other way. In the strict sense, a cognitive map is a “mental representation that has the same basic representational properties and mechanisms as ordinary concrete maps” (Rescorla, 2018, p.35). Put differently, a strict cognitive map is isomorphic with a real map; it is spatially organized in one way or another. A loose cognitive map only encodes the same information. I will refer to the less demanding loose sense from now on. Wiener et al. (2011) place cognitive maps on the third level (spatial constructs).

The step from lower-level skills such as beaconing, route following and path integration, to higher ones such as cognitive maps, is significant. The lower feats rely on an *egocentric* perspective. Animals can beacon with their eyes, ears and noses. They can follow routes by sniffing, feeling, or calling out and listening. Path integration, say by means of step-counting or optic flow, is constrained by the types of bodies and senses animals have. In contrast, complex tools such as cognitive maps imply an *allocentric* perspective. An allocentric view is a view from above or rather from nowhere specifically, on which the organism represents its *own* location in relation to visible and out-of-sight landmarks. Animals (or their brains) need to perform a “coordinate transformation” to attain this perspective (Rescorla, 2018). Some researchers believe that taking novel shortcuts requires coordinate transformation (Bingman, 2011, p.41; see also O’Keefe & Nadel, 1978).^4^ Relatedly, the ability to “move toward a destination without using familiar landmarks” after displacement (Putman, 2021)—sometimes called “true navigation”—also requires extrapolation from sensory input and hence allocentric perspective-taking.^5^

With respect to cognitive maps, neuroscientists have also discovered several cells in the mammalian hippocampus that are correlated with navigation. Place cells respond to specific spatial locations (O’Keefe & Dostrovsky, 1971); head direction cells fire when the mammal’s head is at certain angle with respect to an external reference direction (Taube, 2007); grid cells respond to the local environment, forming a local grid covering that local environment (Hafting et al., 2005) and “Metric information … can be extracted from the firing patterns” (Rescorla, p.37); and finally, regarding border cells, different cells fire when the organism is near different borders (Solstad et al., 2008). Some researchers hypothesize that these cells form the neural underpinnings of a cognitive map, at least in what Rescorla (2018) calls the “loose” sense in that they encode configurational information.^6^

Further support for configurational knowledge seems to come from so-called displacement studies. In displacement studies, animals are relocated from familiar to unfamiliar territory. The big question is, can they return, and if so, how? Animals can’t use familiar cues and so must put their cognitive machinery to work. Rescorla (2018) cites a study by Tsoar et al. (2011) done with Egyptian fruit bats. These bats were moved in a cloth bag 44 km outside their normal flight range, and freed in a large crater (Tsoar et al., 2011). This procedure disabled path integration, beaconing and route-following. Despite initial disorientation, these bats could fly back to their home cave or a familiar feeding site.^7^ Rescorla (2018) concludes that the bats initially needed to orient, given the sensory input available, and then “computed a route to the goal. An explanation along these lines presupposes that bats have a large-scale representation of landmark locations” (p.36). Researchers have carried out displacement studies with many animals, including migratory birds (Thorup et al., 2007; Kashetsky et al., 2021), pigeons (Wiltschko & Wiltschko, 2017; Wallraff, 2005), salmon (DeBose & Nevitt, 2008; Hasler & Scholz, 1983) and crocodiles (Read et al., 2007) with similar results.

The philosophical and empirical research discussed here, is conducted with the premise that organisms will “internalize” their knowledge of the environment. With experience, the structure of the inner world becomes more important and the structure of the outer world less so. Wiener et al. (2011) make no secret of this view. They believe comparative navigation research is most interesting on the levels of spatial primitives and spatial constructs—that’s where functional similarities despite sensory differences can be discovered. They urge that “The challenge for the field [of animal navigation] as a whole is to understand the semantic structure of spatial representations in general, which ultimately entails understanding the behavioral and neural mechanisms by which semantic content is synthesized from sensory inputs, stored, and used to generate behavior” (Wiener et al., 2011, p.51) and that “the hallmark of navigational complexity is the synthesis of internal representations” (p.54). Rescorla (2018) even claims that “Numerous navigational phenomena are difficult or impossible to explain unless we posit cognitive maps in the loose sense. Animal navigation therefore provides strong evidence for a broadly representationalist approach to psychology.” (p.42).

## Against enrichment

Enrichment thinkers will be inclined to say that some of the extraordinary navigational achievements of animals simply can’t be explained without positing cognitive maps or other configurational tools. In other words, complex navigation is “representation-hungry” (Clark & Toribio, 1994). There are, however, at least three related reasons to consider an alternative. All have to do with neglecting the tight coupling between organisms and their environments.

Firstly, the attribution of cognitive maps to nonhuman animals might be anthropomorphic. Wiener et al. (2011) list “external maps, wayfinding signage and human language” on top, whereas on the subordinate level they list, amongst others, cognitive maps (p.53). They tacitly assume that possessing a cognitive map *underlies* map-making. Heft (2013) defends precisely the opposite view: the cultural invention and practice of map-making in humans have made it possible to think in a map-like fashion (see also Ingold, 2000). That is, configurational knowledge—such as map-like thinking—develops within and is sustained by sociocultural practices. Collectively and over generations, humans have invented abstract concepts such as objective space, geometric relations and cardinal directions alongside cultural artefacts such as compasses, maps and satellite imagery. Such inventions make it possible—literally, with the aid of GPS^8^—to look at and reflect on landscapes allocentrically or “from above”.

These inventions, now routinely relied upon by humans, shape individual thinking about the environment during development and throughout life (Heft, 2013, p.277, see also Henrich, 2016, Ch.14). Since children naturally come to participate in social practices pervaded by these inventions, it becomes all too easy to think that map-like, allocentric thought is “natural”, a product of our evolutionary history.^9^ These practices have in a sense become “invisible” to us, and we fail to see how they have shaped and continue to shape map-like thought and allocentric perspective-taking. If Heft (2013) is right about the development of allocentric, map-like understanding, anthropomorphism^10^ looms: we apply a mode of thinking that emerges within a sociocultural niche, and which relies on skilful use of a range of technologies, to other animals who live in vastly different niches and don’t use any of these technologies. In other words, endowing nonhuman animals with map-like thought would result from undue consideration of what our human environment contributes to our abilities.

Secondly, representational or enrichment-based frameworks of navigation devalue the unique bodies and varying niches of nonhuman animals. When perceived navigational complexity increases, on this type of account, the type of tools researcher credit animals with become *less species-specific* and *less environment-dependent.* Wiener et al. (2011) embrace these features rather explicitly, writing that we can think of cognitive maps and other spatial constructs as “being *supramodal* (i.e., independent of or ‘lying above’ specific sensory modality” because they provide semantically [i.e. geometrically] equivalent information about space” (Wiener et al., 2011, p.56). Even more, the authors suggest that supramodality makes meaningful comparisons among species possible. The notion of “supramodality”, however, overlooks the ecological and biological constraints that have shaped the perceptual systems of various animal species. From an evolutionary perspective, it is reasonable to assume that constraints on navigational abilities should continue to hold for “higher” cognitive processes, as they do for “lower” processes. Thirdly, one of the key premises of enrichment thinking is that low-level sensory information is too impoverished to fully explain complex navigation and cognitive processes (Gibson & Gibson, 1955; Gibson & Pick, 2000). However, this assumption has been challenged by ecological psychologists (Gibson, 1979/2015, Gibson & Pick, 2000) and others who draw from their ideas (Kiverstein & Rietveld, 2018; Van Dijk & Withagen, 2016). These scholars argue that environmental information is typically rich and abundant, and animals must learn to detect, differentiate, and utilize the relevant patterns. In fact, the two earlier critiques of enrichment thinking follow logically from this fundamental assumption: when researchers underestimate environmental richness, they overlook the important contributions of the body and the environment to perception and cognition.

## On land: vistas and transitions

Gibson (1979/2015) introduced alternative concepts for navigation with the aim of overcoming simple-complex dichotomies and enrichment-based arguments. He writes that “Neither is adequate. Wayfinding is surely not a sequence of turning responses conditioned to stimuli. But neither is it the consulting of an internal map of the maze, for who is the internal perceiver to look at the map?” (p.189). As alternatives to these behaviorist and cognitivist concepts, he coined the complementary ecological terms *vista* and *transition.*

“A vista is what is seen from here, with the proviso that ‘here’ is not a point but an extended region”, writes Gibson (1979/2015, p.189). Vistas are not things we *look at*, but extended regions that we inhabit or that surround us and in which we look and move around and do things. He adds that “in a terrestrial environment of semienclosed places each vista is unique, unlike the featureless passageways of a maze. Each vista is thus its own “landmark” inasmuch as the habitat never duplicates itself.” (Gibson, 1979/2015, p.189). As an organism travels *through* a vista, a pattern of optic flow is generated that uniquely specifies a route.

As Gibson writes, vistas are almost always surrounded by environmental features that occlude extended views: a tall and wide building, a hill, a dense pack of trees—anything that (partially) occludes another vista. As an organism walks up to and then moves around, over or beneath an occluding feature, a new vista gradually reveals itself at the edge of the vista that is gradually concealed. This optical boundary between the occluding surface and the occluded area is called the *occluding edge* (Gibson, 1979/2015, Heft, 1983, 2019). Gibson (1979/2015) referred to these between-vista changes, specified by occluding edges, as *transitions* (see also Heft, 2013). Transitions “afford looking ahead”, as Heft puts it (Heft, 1983, p.183; Heft, 1996, p.112). They invite an animal to survey the upcoming area.

Crucially, transitions are reversible. The reversibility of transitions plays a critical role in providing navigational information to the organism. By being able to bring into view what has been left behind, and vice versa, the organism is able to accomplish wayfinding in terrestrial environments as a continuous sequence of vistas marked by transitions that uniquely specify a route to a specific destination. Heft (1983) demonstrated that transitions are particularly salient: participants who were shown a video of the transitions along a route were better at finding their way on the actual route afterward and were more confident about their decisions compared to those who were only exposed to a video of the vistas. What’s more, the achievements of the transition-only group were comparable—just slightly worse—to a third set of people who were shown a video of both the vistas and transitions. In addition, his experiments showed that participants became responsive to the order or sequence of the transitions.

An enrichment thinker may suggest that vistas and transitions could provide a *developmental basis* for cognitive maps or other complex representational tools. Tolman (1948), a self-proclaimed “purposive behaviorist” who introduced the concept of cognitive map (being unconvinced by stimulus-response theories of other behaviorists) held a position like this. He believed that cognitive maps are latent effects of extensive exploration, an idea that has taken hold in representationalist thought. This, however, would be a misreading of the ecological concepts. The *information* for animals are not *stimuli* for the sensory receptors, but *patterns* generated by an active and moving organism over time.^11^ Environmental patterns provide a wealth information for wayfinding, but they often escape scrutiny because of a tendency towards enrichment.

## At sea: wind, water and weather

Gibson (1979/2015) and followers were mostly concerned with vision-based navigation on land, but structure over time is also available, for instance, on the open oceans—and not only through vision but also by means of touch, for instance (e.g. brushing of the wind against the skin). In this section, I will discuss the well-known example of the Micronesian and Polynesian navigators of Oceania and their reliance on so-called *etak* segments. Through this example, I will develop some generic concepts for understanding animal navigation without invoking configurational knowledge. These concepts are intended to be broadly applicable while acknowledging the species-specific and niche-dependent aspects of navigation.

About the Polynesian and Micronesian navigators, Hutchins (1995, p.76) writes,

The world of the navigator, however, contains more than a set of tiny islands on an undifferentiated expanse of ocean. Deep below, the presence of submerged reefs changes the apparent color of the water. The surface of the sea undulates with swells born in distant weather systems, and the interaction of the swells with islands produces distinctive swell patterns in the vicinity of lands. Above the sea surface are the winds and weather patterns which govern the fate of sailors. Seabirds abound, especially in the vicinity of land. Finally, at night, there are the stars.

Swell patterns, apparent colors, wind, weather and the whistling of birds; the environment provides much more support than apparent to an untrained eye, ear, nose, or what sense organ have you. Though Gibson (1979/2015) seems to restrict his analysis to navigation on land (which is cluttered with objects), wind, water, weather, and so on, are also *features* of environments that mark the uniqueness of certain locations, and can be used to navigate. The navigational skills of Polynesian and Micronesian navigators, I admit, are tied up with culture and social learning. But the point here is that these navigators don’t transcend their egocentric perspective. They aren’t contemplating how the wind touches their cheeks. They don’t need to “internalize” the information (i.e., stimuli) they gather over time to “construct” routes or maps. Instead, they learn to differentiate among formerly undifferentiated patterns or flows, to selectively attune to relevant patterns given their goals and the circumstances, and where, when and what to do and to attend to (Gibson, 1979/2015; Gibson & Pick, 2000; Szokolszky et al., 2019). That is, they know *how* to get around by generating or amplifying environmental patterns as they sail. Ingold (2000) offers a precise and lively description of how we should imagine their voyages:

Throughout the voyage he [the seafarer] remains, apparently stationary, at the centre of a world that stretches around as far as the horizon, with the great dome of the heavens above. But as the journey proceeds the island of embarkation slips ever farther astern while the destination island draws ever closer. At the same time an island off to one side, selected as a point of reference for the voyage, is supposed to swing past the boat, falling as it does so under the rising or setting positions of a series of stars.

The voyager remains *apparently* stationary as the world—the stars, wave patterns, distant islands—flow by. The way the world flows by is, of course, partially generated by the voyager. Moving ahead, the world flows by not just any one way, but in a way that uniquely specifies the direction and path of travel. Again, the voyagers aren’t pondering, measuring and calculating. They are coordinating their movements with environmental patterns. This perspective, as Ingold (2000) points out, makes sense of an otherwise puzzling fact: these voyagers rely on a reference point, called an *etak*, but the *etak* is usually invisible, if it exists at all:

The fact that the reference island (*etak*) is normally invisible below the horizon, and may not even exist at all, has been a source of puzzlement to many interpreters who – assuming that the mariner’s task is to navigate from one spatial location to another – have proposed that the *etak* is used to obtain a locational fix. […] Rather, pointing to the *etak* is the mariner’s way of indicating where he is in terms of the temporal unfolding of the voyage as a whole […] the Micronesian mariner remembers an inter-island voyage as a sequence of *etak* segments, each of which begins as the reference island falls under one particular star and ends as it falls under the next in line (Ingold, 2000, p.240).

As Ingold makes clear, the *etak* is not a landmark. An *etak* is part of a conglomeration, which may include “The *flow* of waves, wind, current and stars” (Ingold, 2000, p.239). The voyagers keep specific patterns stable during specific legs of the voyage. An *etak* is a mnemonic device, factually summarizing “an immensely variegated terrain of comings and goings, which is continually taking shape around the traveller even as the latter’s movements contribute to its formation.” (p.223). These patterns are inconspicuous to an untrained eye, but “For the experienced inhabitant of this region, the environment is sufficiently differentiated in stable ways to provide some structure” (Heft, 2013 p.281). A seafarer may be prodded by a significant change in the patterning of waves and wind, anchors his gaze to another patch of the night sky and adjust course, sail in some direction for a while, until nudged by the next change.

This example of navigation at sea, I think, illustrates well that “stability” and “structure” don’t always entail concrete objects and surfaces (cf. Ingold, 2011, p.117). As dominantly terrestrial navigators, humans are used to concrete objects (“landmarks”) as features for navigation. But winds come and go, and stars appear on the move throughout the night. Nonetheless, within movement, stability or invariance can be found. Waves, if you know where and how to look, flow by regularly even if not constantly and despite occasional perturbations, small and large. Currents, upwellings and downwellings, for instance, are continuously regenerated and sustained, but they are predictable and regular and hence provide stability and structure.

While specific conglomerations of wind and water flows follow each other reliably and provide structure, waves do not occlude other waves, nor do winds occlude other winds. Unlike in terrestrial navigation there are no occluding edges at sea and hence no transitions in the strict sense. Still, these navigators carve up their journeys in clear and distinguishable *etak* segments, and as one *etak* moves beneath the horizon the navigators gradually enter into the next *etak* segment. Obviously, learning to perceive these patterns and discover these structures asks for hands-on experience—but they are out there to be exploited, so to speak. I will adopt Ingold’s more general, less vision-based term “segment” to signal similarity, though not identicality, with vistas, and for the same reason, use “shift” instead of “transition”. The *etak*-based navigational technique is just one example of wayfinding rooted in segments, shifts and sequences. It is a localized interpretation that is developed in response to distinct environmental patterns within a specific niche. These terms are equally valuable for comprehending nonhuman animal navigation from an organism-environment relational perspective.

Accordingly, I suggest the following way forward: If we wish to explain animal navigation without invoking configurational knowledge, we could focus on identifying (i) segments, which are specified by environmental patterns; (ii) shifts, which are the relatively larger changes *between* segments; and (iii) sequences, which are higher-order sequential patterns of segments and shifts. All of these environmental structures are detectable over time, and only over time. And while there may be substantial variation in segments and shifts depending on the coupling between the kind of animal (given its particular perceptual systems) and the structure of the environment, these concepts offer a general approach to address questions about complex feats of navigation without invoking configurational knowledge.

## The segments, shifts and sequences of the scentscape

In this section, I will delve into the topic of olfactory navigation by albatrosses over the vast open oceans.^12^ Despite the seemingly unpredictable nature of this environment, we can observe unique segments, inter-segment shifts, and discernible structural information over time.^13^ I will use the case of the albatross to exemplify a broader point: that despite a sometimes apparent lack of structure, the environment usually offers a remarkable degree of stability and structure. Accordingly, discovering patterns and structure in unexpected places can help us develop a less human-centered understanding of how animals navigate their environments.

**Fig. 2:**
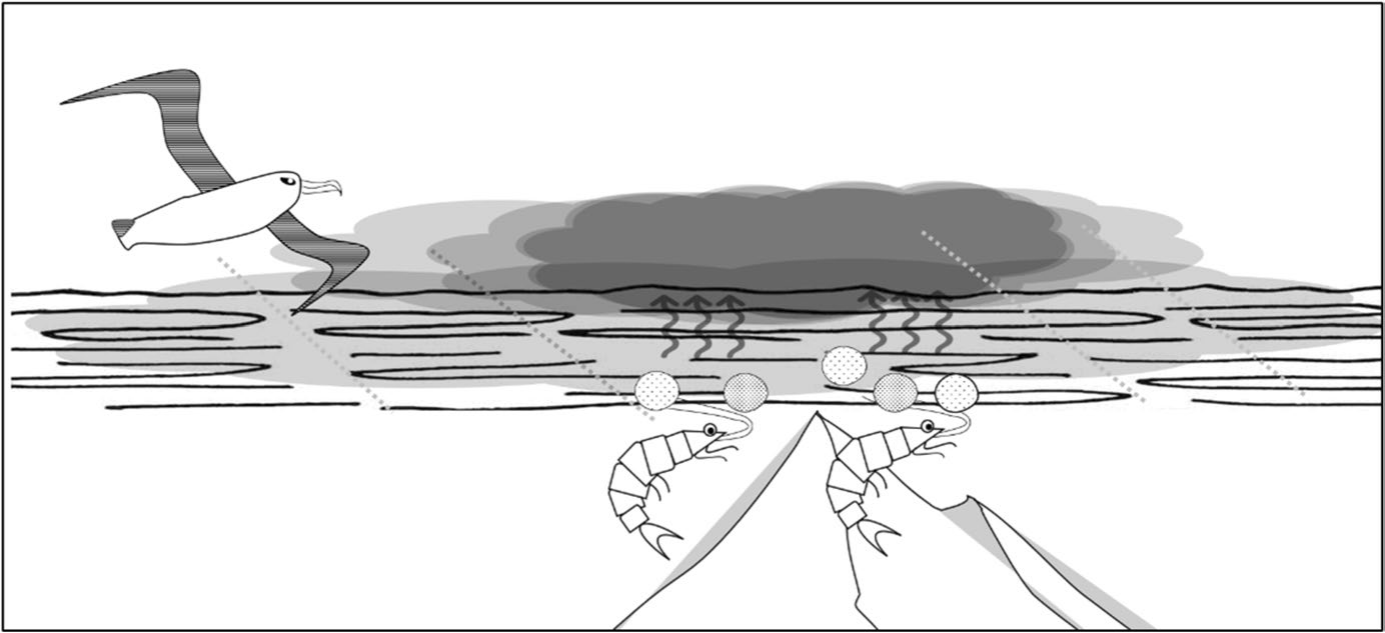
On large spatial scales, concentrations of dimethyl sulfide (DMS) reflect seabed topography, providing structure and stability for navigation over the open oceans. The cloud signifies high concentrations of DMS: concentrations are higher at the location of the submerged mountain. The dotted lines signify a shift between a segment of relatively stable (low) scent levels, a segment of increase, a segment of relatively stable (high) scent levels, a segment of decrease, and another segment of relatively stable (low) scent levels

Albatrosses cover hundreds of kilometers over the oceans and *smell* the oceans to stay oriented. Initially, odor may seem to lack the structure that is required for navigation. Due to the dynamics of odor dispersal, the concentration of scents can be patchy and irregular, rather than forming smooth gradients (Nevitt et al., 2008, p.4576). The process of scent-based navigation for albatrosses is far more intricate than simply homing in on a loud sound, following a trail, or sensing a gradient. One of the odors that albatrosses rely on is referred to chemically as dimethyl sulphide (DMS) and smells fishy or sea-like to us. DMS is produced when zooplankton (such as krill, which albatrosses eat) graze on phytoplankton; phytoplankton excrete a chemical precursor of DMS which rapidly converts into DMS. As a consequence, areas with higher concentrations of phytoplankton have higher concentrations of DMS (see Fig. 2).

Interestingly, researchers have found that DMS concentrations over large spatial scales—stretching thousands of square kilometers—vary with the topography of the seabed, and that concentrations are particularly high around seamounts, because seamounts create upwellings and currents that bring nutrients to the surface, which allow phytoplankton to grow. Hence, the ocean provides an odor structure that albatross can use to navigate (Nevitt, 2000, 2008; Nevitt & Bonadonna, 2005).

To specify, albatross navigation (i) consist of segments, which are specified by environmental patterns. That is, each segment (“what can be smelled from here”) smells unique and moving through a segment generates a unique pattern of olfactory flow. The scent-based segments are similar to vistas in that they have different properties, such as varying levels of scent, whether these levels increase or decrease or are relatively constant, and how much they do. These segments—increase, decrease, constancy—are invariant structures within the constant flux of scent. Secondly, there are (ii) shifts, which are the relatively larger changes *between* segments. For instance, a shift from “relatively constancy” to “major increase”, or from “major decrease” to “minor decrease”. These are not aptly called *transitions* for lack of occluding edges, but the environmental, olfactory structure is such that the scentscape is heterogenous and variegated, rather than homogenous and evenly distributed—hence, there will be relatively large, more drastic, olfactory changes, as compared to the smaller shifts one may find within a segment. These shifts too are higher-order invariant structures, as they are, effectively, larger changes in patterning within smaller changes in patterning, and so these too are only detectable over time. Thirdly, there are (iii) sequences, which are higher-order sequential patterns of segments and shifts. As in the case of terrestrial navigation by vision, albatrosses are able to find their way around through sequences of segments and shifts that uniquely specify routes to destinations. Instead of unique paths specified by a series of vistas and transitions, however, knowing as an albatross where you are and where you are going, is a series of increments, decrements and relative constancy—for instance, *minor increase-increase-constant(high)-slight decrease-constant(low)-increase-decrease—*towards a distal resource.

My albatross example is meant to illustrate a general point: wayfinding is bound up with the perceptual systems and environmental niches of animals. To the extent that we can speak of “similarities”, we won’t find them in similar “internal” capacities, but rather in ways that animals exploit persistent environmental features. For instance, other species such as certain fish, harbor seals and whale sharks also exploit underwater structures by means of DMS (Nevitt, 2008, p.1707)—even if segments and shifts for these animals may not map *exactly* onto each other, given differences in their perceptual systems. Compared to olfactory navigation over the oceans or visual navigation on land, navigating *in* water, *through* the air or *beneath* the surface—be it by touch, vision, hearing or any other perceptual system—will be different again; segments and shifts will be specified differently and uniquely depending on the organism-environment system under investigation.

## Goal directedness without goal representation

Can the ecological view, which emphasizes the gradual uncovering of environmental structure over time, truly make cognitive maps and other configurational tools in nonhuman animals obsolete? One remaining question seems to be: wouldn’t animals need to know where they are going in advance, and does that not imply that they represent their destination? The possibility of goal-directedness without goal-representation is difficult to fathom within an enrichment paradigm. This difficulty explains why even Nevitt (2008) speculates that albatrosses “build up a map of these [oceanic] features over time” (p.1707), despite the richness of information in oceanic structures that she has discovered.^14^ The ecological approach renders configurational knowledge redundant by appealing to the *hierarchically nested structure* of the environment.

**Fig. 3:**
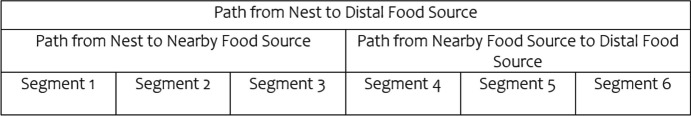
This table is an adaptation of the one provided by Heft (1996)

Gibson (1979/2015) wrote that, “for the terrestrial environment, there is no special proper unit in terms of which it can be analyzed once and for all. There are no atomic units of the world considered as an environment. Instead, there are subordinate and superordinate units.” (p.5) For instance, an apple is nested within a branch, which is nested within the tree, which is nested within the wider landscape, and so forth. We find this nested organization in navigation too, where “a particular series of vistas would be nested within some higher-order unit” (Heft, 1996, p.118), as in Fig. 3: Segment 1, 2 and 3 are nested within the superordinate “Path from Nest to Nearby Food Source”, segment 4, 5 and 6 are nested within the superordinate “Path from Nearby Food Source to Distal Food Source”, and these two superordinate units are nested again within the superordinate “Path from Nest to Distal Food Source”.

The environment’s nested structure is reflected in the activity of organisms; it too can be described as a “temporally-structured, hierarchically-nested event” (Heft, 1996). For instance, a tree affords climbing, which affords reaching higher branches, which affords gathering apples, and hence when we stand in front of the tree we perceive the apples as “gatherable”—even if the apples are occluded by the surrounding leaves and you can’t directly see them. The activity of gathering the apple from the tree is temporally extended and hierarchically nested. The activities of walking to the tree, climbing it and picking the apple are activities for a particular organism that, with experience, have become nested within, and subordinate to, the superordinate activity of “gathering the apple”. In this example, activity is goal *directed* without goal *representation.* The activity is fully situated and embodied, and can be explained by referring to the nested structure of the environment.

Similarly, in the case of navigation, an animal may initially limit its search to a nearby food source. On subsequent journeys, the animal will venture farther. The environmental patterns, specifically the shifts, will come to offer opportunities for *further exploration* for that organism, in addition to the resources found within that segment. With experience, then, subordinate activities (e.g. locating the nearby food source) will have become nested within superordinate activities (e.g. finding the way to the distal food source). Similar to how a terrestrial animal can learn to perceive the occluded apple as gatherable, an albatross, for instance, can learn to perceive that plenty of fish are edible even if the albatross can’t currently smell them (see also Van Dijk & Withagen, 2016, Kiverstein & Rietveld, 2018; Van Woerkum, 2021). What’s more, since there’s no proper unit of analysis, there’s no principled limit to these abilities, as each superordinate activity (e.g. to the distal food source) can itself become subordinate (e.g. to the activity of finding an even more distal food source).

Here, again, activity is goal directed without goal representation. Animals are able to find their way by exploiting environmental structure. At no point do they need to combine isolated aspect of the environment and calculate their direction and distance to their destination *before they go* (i.e., configurational knowledge). Instead, nonhuman animals can gradually determine their destination *as they go along* (see Van Dijk & Rietveld, 2018). They learn to perceive environmental structure over time and become attuned to segments and shifts *as they occur*. So, while animals can engage with affordances beyond what can be perceived through sight, smell, or touch in their immediate surroundings, this must not be thought of a representational, or as implying configurational knowledge.

## Conclusion

Enrichment thinkers, such as Wiener et al. (2011) argue that as perceived navigational complexity increases, the organism becomes more and more detached from the environment. As far as their theories are concerned, the details of concrete situations that animals navigate *through* are barely relevant—what matters is whether there is *something* to see, hear, smell, or in more complex cases, calculate, visualize, think. In this paper, I have offered an alternative for the hypothesis that navigational complexity should be accompanied by internal (representational) complexity. The alternative, that animals become responsive to segments, shifts and sequences—information about structure detectable over time—through processes of perceptual learning, avoids anthropomorphism (such as ascribing configurational knowledge) and respects biological and ecological constraints (by taking all navigational skills and tools to depend on transactions between an animal’s perceptual systems and concrete ecological circumstances).

An ecological approach also yields implications for empirical research. The discovery of structures that animals rely on (i.e., segments, shifts and sequences), the emergence and persistence of environmental structure, and the streamlining of navigation with experience due to perceptual learning, are ecological phenomena that are foregrounded by an ecological approach. Such ecological circumstances are at least equally important as sensory mechanisms, since two animals of the same species in different environments may well use different perception-action strategies precisely given that they have to rely on different environmental structures. An ecological approach also demands a sensitivity to scale (what could be a segment and shift for a desert ant?) and embodiment (how can the animal gage the structure with its specific body and perceptual systems?). Moreover, investigating similarity (such as reliance on particular environmental structures, such as DMS in several marine creatures) and diversity (how these marine creatures may, because of their different bodies and ways of living, nonetheless be immersed in different segments and shifts) are equally important and valuable. These focus points follow naturally when wayfinding or navigation abilities are tied to, or constrained by, the perceptual systems of animals and the particular niches they occupy.

Finally, an ecological approach heightens awareness of the ecological relations that enable animal wayfinding (see also Van Dijk, 2021). As we saw, the ability of albatrosses to find their way around depends on what other creatures in that same environment are up to. Consequently, changes in the presence and activity of zooplankton related to oceanic acidification due to increased CO_2_ concentrations in the atmosphere, may eventually impact the structures that albatrosses depend on to navigate (Hammill et al., 2018). Not least because of its pragmatist roots, any ecological psychology that lives up to its name—being truly *ecological*—tries not only to understand isolated phenomena—such as how a particular animal finds its way around—but also how living and acting organisms, including us, are tangled up with each other in particular environments so as to enable and maintain, or instead, disable and distort these navigational abilities.

## Data Availability

Not applicable.

## Footnotes

1 I’m paraphrasing E. J. Gibson and Pick (2000, p. 150) here, who strongly oppose this type of view.

2 Gibson used the term ‘wayfinding’ for his theory, as do for instance Heft (2013) and Ingold (2000) who build on Gibson’s work. I use the terms ‘wayfinding’ and ‘navigation’ here interchangeably to make clear that this debate is about animal navigation, not something (slightly) different.

3 Similar issues are found in the mindreading and episodic-like memory debate in animal cognition, where what counts as true mindreading or genuine episodic memory remain contentious topics due to higher-lower cognition dichotomies that are often implicit in representationalist approaches to these topics (see Van Woerkum 2021, 2022).

4 Interestingly, Rescorla (2018) herself mentions that there’s also evidence that animals often do not use the shortest routes to return. This seems in line with the ecological approach, that predicts animals to normally follow *meaningful routes* (i.e. routes that pass through important resources) rather than the shortest routes (Heft, 2013, see also Sects. 3 and 4).

5 A predominant theory for ‘true navigation’ is the gradient hypothesis. Since animals are displaced outside their familiar range, they must extrapolate from what they know about environmental gradients, such as geomagnetic field gradients (Kishkinev et al., 2021) or odor gradients (Bingman, 2011). Whether gradient sensing is sufficient as a mechanism for cognitive maps remains a topic of debate (see Lohmann et al., 2007), but not one I will delve into here.

6 These discoveries are not sufficient to support the cognitive map hypothesis: an ecological approach turns the tables on this claim and says that the structure of the environment, through the animal’s explorations, organizes the firing patterns of these neurons, rather than the other way around. However, an investigation of these findings is beyond the scope of this paper (but see Bruineberg & Rietveld, 2019; see also Hutto & Myin, 2017).

7 Where they would fly to, depended on whether they were hungry or not.

8 At the same time, using GPS can decrease sensitivity to environmental patterns (see Heft et al., 2021), decreasing the ability to learn specific routes.

9 Danziger (2008, p.4) makes a similar point with regard to memory.

10 Or what could better be called WEIRDocentrism: applying concepts employed by people in Western, educated, industrial, rich and democratic societies (Henrich, 2020) to non-WEIRD beings.

11 When I use the term information in the context of ecological psychology, I’m referring to environmental patterns that are generated (or at least amplified) by an active and moving organism.

12 For good measure, wayfinding is likely to be a multisensory, situated endeavour, constrained by what information is available to specify environmental structure rather than by any specific sensory mechanism (see Cheng & Newcombe, 2005, Heft, 2013, p.287). Albatrosses may additionally exploit patterns of wind and water, other types of smells, and perhaps geomagnetism (Wynn et al., 2020) and infrasound (Patrick et al., 2021), but I will focus on smell here. See also Stoffregen et al. (2017) who are against individuating perceptual systems, and argue that we should consider the senses as *one* perceptual system.

13 Gibson (1966) wrote about smell, but mostly about odors directly emanating from a source, such as food, predators or mates—though he also mentions Hasler’s work, if only in passing, on how salmon are able to find their way back to their natal stream by relying on their sense of smell (see e.g. Hasler & Scholz, 1983).

14 As Heft (1996) writes, representational explanations may also be, in large measure, logically unnecessary after one has articulated a sufficiently rich description of the environmental information available to be perceived.
